# Launching a multidisciplinary European collaboration towards a cure for HIV: The EU2Cure Consortium

**DOI:** 10.1016/j.jve.2021.100045

**Published:** 2021-05-23

**Authors:** C. Rokx, H.A.B. Prins, L. Vandekerckhove, S.J. Fidler, J. Frater, M. Bracchi, O.S. Søgaard, M. Tolstrop, T.A. Rasmussen, M. Salgado, J. Blanco, J. Martinez-Picado, B. Clotet, G. Tambussi, A. Groenendijk, A. Verbon, C.A.B. Boucher

**Affiliations:** a Erasmus MC University Medical Center, Netherlands; b Ghent University, Belgium; c Imperial College London, United Kingdom; d University of Oxford, United Kingdom; e Chelsea and Westminster Hospital, United Kingdom; f Aarhus University Hospital, Denmark; g IrsiCaixa Barcelona, Spain; h San Raffaele Scientific Institute Milan, Italy

**Keywords:** HIV cure, Collaboration, Network, Reservoir, Shock and kill, HIV cohort

## Abstract

We felt the urgency to launch the EU2Cure Consortium to support research and find a cure for the human immunodeficiency virus (HIV) infection through intensified collaboration within Europe. This consortium is open to stakeholders on cure in Europe from academia and the community to connect. The aim of this consortium is to intensify the research collaboration amongst European HIV cure groups and the community and facilitate interactions with other academic and community cure consortia, private parties, and policy makers. Our main aim is to create a European research agenda, data sharing, and development of best practice for clinical and translational science to achieve breakthroughs with clinically feasible HIV cure strategies. This consortium should also enable setting up collaborative studies accessible to a broader group of people living with HIV. Besides reservoir studies, we have identified three overlapping scientific interests in the consortium that provide a starting point for further research within a European network: developing “shock and kill” cure strategies, defining HIV cure biomarkers, and connecting cure cohorts. This strategy should aid stakeholders to sustain progress in HIV cure research regardless of coincidental global health or political crises.

Almost five decades into the human immunodeficiency virus (HIV) pandemic, a cure has still not been achieved. Recent overviews from the field indicate that HIV cure research should be conducted in more diverse settings and benefits from timely defining target product profiles.^1^, ^2^, ^3^ These should contain key parameters such as the efficacy, toxicity and necessary infrastructure of potential future cure interventions for which research and early stakeholder engagement are essential. We fully agree with this perspective and would like to contribute with a joint action to materialize this strategy. Next to existing national and international HIV cure study networks (e.g. EPIICAL, https://www.epiical.org/; ACTG, https://actgnetwork.org/studies/; IAS-Cure, https://iasociety.org/hivcure; CHERUB, https://www.cherub.uk.net/;) and the more recently launched Dutch NL4Cure and African HIV Cure Africa Acceleration Partnership (HCAAP) initiatives),^3^^,^^4^ we believe that there is a need to establish a complimentary European HIV Cure Research Consortium which has been named EU2Cure. This network will help to intensify collaborations primarily amongst Europe’s HIV cure research groups. Establishing a European-wide framework is crucial to better advance HIV cure research rather than working as individual groups. Such a network also promotes connections to other international cure initiatives from either academia, the community, and private parties including industry. It supports the capacity building to perform state of the art fundamental research and offers the possibility for well-powered clinical interventional HIV cure studies aimed at generating breakthroughs. Collaborative studies will be accessible to a broader and more diverse population and allow for the streamlining of studies between groups, thereby leading to a more efficient research strategy. This will also benefit the study participants who are willing to take part in these studies.

The EU2Cure Consortium builds upon a successful experience with earlier collaborative HIV research initiatives in Europe. It also adds a new perspective within Europe, which has not yet been covered by other successful European initiatives on therapeutic HIV vaccinations (EHVA, https://ehv-a.eu/) or stem-cell transplantations (IciStem, https://www.icistem.org/). A live kick-off meeting on the 14^th^ of June 2019 (Erasmus University Medical Center, Rotterdam, the Netherlands) and several virtual meetings during the global COVID-19 emergency have strengthened our purpose and aims. Here, we provide our vision for the near and more distant future of clinical and translational HIV cure research in Europe.

We see three major topics that will be our initial collaborative focus. This is based on the consortium members’ overlapping interests and expertise: developing “shock and kill” cure strategies, defining HIV cure biomarkers, and connecting cure cohorts. We presently only have limited options to restrict the HIV reservoir as its size can be significantly impacted by initiating antiretroviral therapy (ART) early in patients with acute HIV infection or by interventions aimed at decreasing an established reservoir, to a level allowing the interruption of ART. Reports on successful strategies are very limited and only reported in a few people who underwent allogeneic stem cell transplants (SCT) for hematologic malignancies, with matched donors harboring homozygous deletions in the genomic CCR5d32 region.^5^^,^^6^ Due to a high risk of complications and death, SCT is not a safe, feasible or realistic path to a cure for all patients. Alternatives to SCT to achieve viral control are scarce but subject to intense research globally and set as a priority within our consortium. Emerging from this vision, we see synergy in our efforts to develop new cure strategies.^7^, ^8^, ^9^, ^10^, ^11^, ^12^, ^13^ Step by step we and others have advanced the field by exploring new ways to disrupt HIV latency with drugs such as HDAC inhibitors romidepsin, panobinostat or vorinostat alone or in combination with immunomodulatory agents in studies such as in the RIVER or REDUC trials, or by using BAF inhibitors as in the LUNA trial.^7^^,^^8^^,^^12^ Pioneering interventional studies on “shock and kill” are still relatively small, often lack control groups, and have limited research material for analysis. Further unravelling of the composition of the latent HIV reservoir should also be a central research theme to assess the successful efficacy assessment in interventional studies. Significant progress has been made, including by EU2Cure members, in the understanding of proviral latency at genomic levels, e.g. by the identification of nucleosome positioning over the HIV promoter, methylation patterns, and role of 7SK snRNP disruption in HIV transcript elongation.^14^, [15], 16 Upon analytical treatment interruption in the HIV-STAR study, rebounding virus was shown to consist of genetically identical viral clones, originating from diverse reservoir sources, while treatment interruption within the SPARTAC trial highlighted the issue of T cell exhaustion in post-treatment control.^17^, ^18^, ^19^ The interplay between all the factors involved in reservoir maintenance and viral rebound is complex and incompletely understood. When conducting cure studies, researchers have to deal with the rarity of proviral latency in CD4^+^ T cell (sub)populations, which can reside in different, often difficult to target, anatomical sites. This makes reservoir studies challenging, expensive and requiring large sampling volumes. Biomarker identification of the reservoir size with successful viral control is therefore the second main topic within the consortium since it would simplify patient selection and readout of interventional studies. These examples underscore that working together will help to maximize the output of our complementary studies such as systematic meta-analyses of available data, sharing best practice to allow assay optimization, and optimal use of available study samples.

Cohort studies are set as a third priority. An effective strategy is to limit the size of the HIV reservoir by introducing ART in the acute stage of the infection. These early treated individuals are attractive for cure interventions due to their much smaller reservoirs and a relevant subset of these patients seem to be able to achieve post-treatment control, even without additional interventions.^20^ Our consortium researchers currently are all individually involved in acute HIV cohorts from 5 European countries (eCLEAR, INACTION, HEATHER, ACS, NOVA) with already over 1000 participants, often with in-depth sampling, including leucapheresis or sigmoidoscopy. The majority of patients worldwide are nonetheless still diagnosed and treated during the chronic stages of the infection. An interesting observation from the LoViRet cohort has shown that a relevant proportion of treated patients who started ART during chronic infection harbor reduced levels of latent reservoirs.^21^ The limited number of available samples precluded further analysis to unravel the drivers behind this observation, highlighting the need for additional cohort studies. One such example within our consortium is the CHRONO study, where longitudinal follow-up is combined with extensive sampling by leucapheresis of individuals who initiated ART during the chronic stages of infection. These cohorts are clear examples where collaboration at the European level offers unique possibilities and can also benefit the global research field. This allows more in-depth reservoir studies with increased sample availability and power to search for biomarkers of viral control as well as to test hypotheses on putative curative compounds in interventional studies. Linking cohorts, both with acute and chronically HIV infected patients, which are inclusive in terms of backgrounds and sexes, increases the capacity to study research questions in these relevant patient groups.

To be able to better determine and coordinate future directions of cure studies across Europe, we have initiated the EU2Cure Consortium to link key European clinical and translational scientists in the cure research field and invite interested parties to join us. In the future we will endeavor to conduct larger collaborative cohort and interventional studies. By enhancing in parallel knowledge exchange within Europe, these studies should also lead to more tailored and personalized, cure treatments. This optimization of the scale and speed of recruitment for novel interventional clinical studies is another promising outcome where a cross-European cooperation can greatly facilitate unique research opportunities. An added advantage offered by the European setting is that many national health systems in Europe share the possibility to integrate care with research, allowing nationwide recruitment and follow-up in most settings.

With an increasing number of HIV cure studies worldwide,^22^ combining the experience from trials on the same topic can help direct future European trials and facilitate harmonizing study designs and data collections. A clear ambition of our consortium is to connect these research networks. Second, since strategies towards an HIV cure are expected to use more complex and individualized interventions, we also see our consortium as a help in the initiation and coordination of such studies, including the regulatory aspects across Europe. Therefore, these studies will provide a larger critical mass by connecting more experienced senior researchers and talents from the next generation of HIV cure scientists. Furthermore and importantly, as EU2Cure Consortium researchers we feel that we should not close our eyes to the changing political and societal views towards HIV over the last decade. In several European countries, in line with global trends, the political systems has increasingly increasingly moved towards more conservative opinions on sexual freedom and restrictions of the right for HIV preventative measures, abortion or same-sex intimacy. These directly affect many of our patients mentally and physically and can also threaten HIV care and research initiatives, further aggravated by global crises such as the current COVID-19 pandemic. We believe that one way to overcome this is to act together for a strong European scientific agenda on HIV cure that is both durable and able to deliver a synergistic output. Developing a EU2Cure research agenda with stakeholders from the clinical and translational scientific field and patient community associations across Europe on HIV cure research will help keeping HIV on the radar, benefitting those who need a solution to the HIV/AIDS pandemic most, and therefore remains a priority.

It is important to highlight that the current EU2Cure Consortium only represents a starting point. We would like to invite other European HIV cure research groups to consider contacting us in order to strengthen it. We also explicitly invite European patient community stakeholders on cure to connect with us to guarantee the meaningful and timely involvement of the HIV community and to help setting up ethical standards for HIV cure research. Our efforts as a joint European venture are ultimately driven by the need for a world without HIV in which all parties, including Europe, can in hindsight say that they have strongly contributed to this goal.

## Funding

This collaboration is supported by the Dutch AidsFonds (grant number: P-60604).

## Declaration of competing interest

The authors declare that they have no known competing financial interests or personal relationships that could have influenced the work reported in this paper.
